# Induction of Drug Transporters Alters Disposition of Risperidone - A Study in Mice

**DOI:** 10.3390/pharmaceutics2020258

**Published:** 2010-06-02

**Authors:** David Holthoewer, Christoph Hiemke, Ulrich Schmitt

**Affiliations:** Department of Psychiatry and Psychotherapy, University Medical Center of the Johannes Gutenberg-University Mainz, Untere Zahlbacher Strasse 8, 55101 Mainz, Germany; E-Mails: hiemke@uni-mainz.de (C.H.); schmitt@psychiatrie.klinik.uni-mainz.de (U.S.)

**Keywords:** drug transporter, P-glycoprotein, risperidone, 9-hydroxyrisperidone, antipsychotics, disposition

## Abstract

Pharmacokinetic interactions, e.g. modulation of drug transporters like P-glycoprotein at the blood-brain barrier, can be a reason for treatment non-response. This study focuses on the influence of induction of drug transporters on the disposition of the antipsychotic drugs risperidone and 9-hydroxyrisperidone. Brain and serum concentrations of risperidone and its active metabolite 9-hydroxyrisperidone, which are known P-glycoprotein substrates, were measured after drug transporter induction with rifampicin, dexamethasone or 5-pregnene-3beta-ol-20-on-16alpha-carbonitrile using high performance liquid chromatography. Disposition of risperidone and 9-hydroxyrisperidone was dramatically decreased in mouse brain and serum after drug transporter induction. The metabolism of risperidone was also affected.

## 1. Introduction

**Figure 1:**
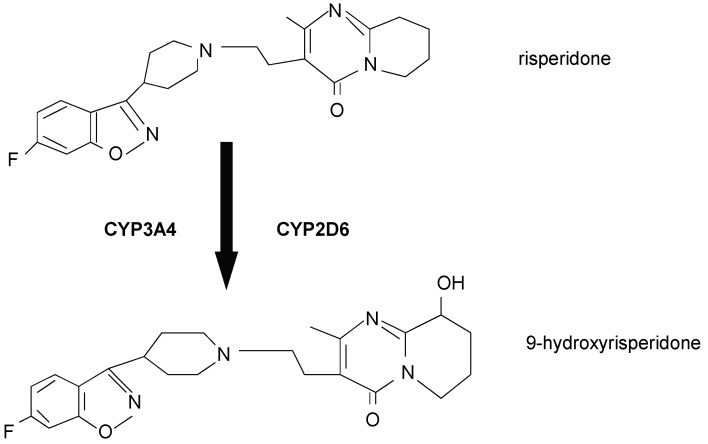
Metabolism of risperidone in humans [10].

The blood-brain barrier (BBB) regulates the distribution of drugs in the central nervous system (CNS). To reach their target, CNS active drugs need to overcome this barrier. P-glycoprotein (P-gp), among other transport proteins, is a widely expressed efflux transporter in cerebral endothelial cells. It is a 170 kDa protein and belongs to the adenosinetriphosphate binding cassette (ABC) superfamily. These transporters are highly expressed across species [1]. The expression and function of P-gp is controlled by the mdr1a/1b genes in mice and the ABCB1 gene in humans [2]. P-gp influences the disposition of various drugs [3]. There is no clear structure-affinity relationship for substrates of P-gp or other drug transporters. *In vitro* and *in vivo* studies demonstrated that a number of psychoactive drugs, like antidepressants and antipsychotics, are P-gp substrates [4,5,6,7,8]. For *in vivo* investigations P-gp wildtype and double-knockout mice (mdr1a/1b -/-) are commercially available. By using this *in vivo* model, the impact of P-gp on brain concentrations of different kinds of psychoactive drugs, including state of the art antipsychotics, has already been demonstrated [7,8,9]. Atypical antipsychotic drugs are recommended as first line treatment in the therapy of schizophrenia. In two animal *in vivo* studies risperidone and its active metabolite 9-hydroxyrisperidone, also called paliperidone, showed significant changes in their disposition when wildtype mice were compared to P-gp knockout mice [7,9]. In both studies, 9-hydroxyrisperidone showed increased P-gp affinity compared to risperidone. As a conclusion, risperidone and 9-hydroxyrisperidone are both substrates of P-gp and can be used as model substrates for the evaluation of drug transport at the BBB. Quite interestingly, behavioral consequences of P-gp expression were observed in these mice, which indicated the role of this transporter in pharmacokinetic interactions due to modulation of its efflux transport activity, which can influence the efficacy of risperidone treatment [9]. The therapeutic effect of CNS active drugs can be limited by enforced efflux transport and this could be one of the reasons for treatment non-response or limited therapy response. In humans, risperidone is metabolized into 9-hydroxyrisperidone by cytochrome-P450 (CYP) enzymes CYP2D6 and to a lesser extent by CYP3A4 (Figure 1) [10].

Metabolism of risperidone in mice has not been further investigated yet. Metabolites, other than 9-hydroxyrisperidone were not detected with the reversed phase high performance liquid chromatography (HPLC) method used in this study and were not included in the validation process. Therefore, other possible metabolites have not been considered in this study. Previous investigations could demonstrate that both risperidone and 9-hydroxyrisperidone concentrations could also be analyzed in brain and serum samples of mice, despite other possible metabolic pathways [9,11]. This study is aimed to demonstrate the clinical importance of enforced drug transporter expression by treatment with the known drug transporter inducers rifampicin, dexamethasone and 5-pregnene-3beta-ol-20-on-16alpha-carbonitrile (PCN) in mice, and the consequences of increased brain efflux on the pharmacotherapy of psychiatric diseases. For characterization of drug transporter activity, mice brain and serum concentrations of risperidone and 9-hydroxyrisperidone were analyzed using an established HPLC method [9].

## 2. Materials and Methods

### 2.1. Drugs

Risperidone solution for injection (Risperdal®) was obtained from Janssen-Cilag GmbH (Neuss, Germany) and rifampicin-sodium (Eremfat®) from Fatol GmbH (Schiffweiler, Germany). PCN and corn oil (used as solvent) were purchased from Sigma-Aldrich (Steinheim, Germany). Physiological saline solution 0.9% was received from Braun (Melsungen, Germany). Risperidone, used as control for the HPLC analysis, was purchased from MP Biomedicals (Illkirch, France); 9-hydroxyrisperidone, used as control for HPLC analysis, was kindly provided by Janssen-Cilag (Beerse, Belgium). Methanol (HPLC grade) and dexamethasone-21-di-sodium-dihydrogen-phosphate (Fortecortin® inject) were supplied from Merck (Darmstadt, Germany). Isoflurane for anaesthesia (Forene®) was purchased from Abbott GmbH & Co. KG (Wiesbaden, Germany).

### 2.2. Animals

A total of 90 male FVB/N mice (25–45 g; P-gp status mdr1a/1b +/+) from the animal facility of the University Medical Center were used (n = 15 per group; 5 mice for each investigated time point). Animals were housed in groups of 2–5 with free access to food and water. A 12-h light–dark cycle was maintained at a temperature of 22 °C and a relative humidity of 60%. All experiments were conducted in accordance to the official regulations for the care and use of laboratory animals and approved by local authorities.

### 2.3. Study design and drug administration

Mice, n = 15 per group, were divided into 5 groups (rifampicin, dexamethasone, saline control, PCN, corn oil control). Rifampicin, dexamethasone and PCN were injected intraperitoneally (i.p.) for 4 days consecutively. Rifampicin was used at a dose of 10 mg/kg/d, dexamethasone at a dose of 50 mg/kg/d and the known murine selective pregnane X receptor (PXR) activator PCN at a dose of 25 mg/kg/d for drug transporter induction [12,13]. As controls, mice were treated with solvent only. In the rifampicin and dexamethasone groups, controls received physiological saline, and PCN control groups were treated with corn oil, which was needed as a solvent because PCN is highly lipophilic and insoluble in aqueous solutions. Drug transporter inducers were injected once daily at a 24 h interval. On day 5, risperidone was injected i.p. at a dose of 3 mg/kg. 1, 3 and 6 hours of after injection of risperidone, 5 mice were anesthetized and decapitated, respectively. Trunk blood and brain tissue samples were collected and the concentration of risperidone and its active metabolite were measured using an established HPLC method [9]. The sum of risperidone and 9-hydroxyrisperidone concentrations were also calculated as active moiety because both exhibit similar receptor profiles and therapeutic efficacy [14,15]. Risperidone active moiety is used in the clinical routine of therapeutic drug monitoring (TDM) to evaluate pharmacological treatment of psychiatric diseases. The risperidone/9-hydroxyrisperidone ratios (RIS ratios) were calculated to clarify effects of the drug transporter inducers on metabolism of risperidone. Brain/Serum ratios of risperidone and 9-hydroxyrisperidone and of risperidone active moiety were calculated to illustrate changes in the distribution of risperidone and 9-hydroxyrisperidone. 

### 2.4. Sample analysis

Sample preparation and analysis were adjusted to a previously published and established method [9]. The method uses HPLC on-line solid phase clean up and column switching by chromatographic analysis and spectrometric detection. For sample clean up, Perfect Bond CN (5 μm, 10 × 4 mm) was used. Chromatographic separation is conducted on a Hypersil ODS C18 column (5 μm, 150 × 3 mm). The method was linear in a range from 2–60 ng/mL with a detection limit below 1 ng/ml for risperidone and 9-hydroxyrisperidone, with a precision of <3.6% (risperidone) and <1.1% (9-hydroxyrisperidone), and an accuracy of <3.0% (risperidone) and <4.7% (9-hydroxyrisperidone).

### 2.5. Statistical analysis

Statistical comparisons between groups were carried out using SPSS version 17.0 (SPSS GmbH Software, Munich, Germany). Student’s *t*-test was used when Gaussian distribution was assumed with the Shapiro-Wilk test. Otherwise, Mann-Whitney U-test was used to assess the statistical significance of the differences between the results. Differences between related parameters were considered to be statistically significant for p values less than 0.05.

## 3. Results and Discussion

### 3.1. Brain concentrations of risperidone and 9-hydroxyrisperidone

**Figure 2:**
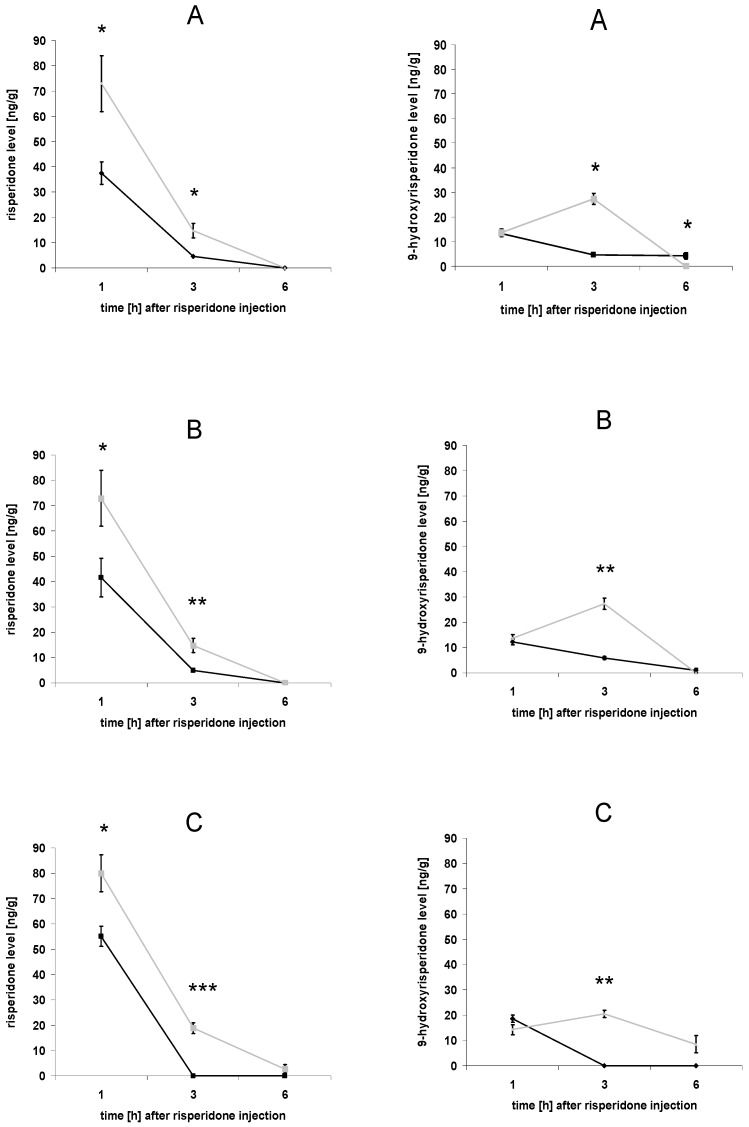
Brain concentrations of risperidone and 9-hydroxyrisperidone. Brain concentrations of risperidone (left side) and 9-hydroxyrisperidone (right side) after induction of drug transporters with 10 mg/kg/d rifampicin (A), 50 mg/kg/d dexamethasone (B) or 25 mg/kg/d; PCN (C) *vs.* controls for 4 days. Drug transporters induced mice = black lines, and controls = grey lines. Data presented are mean +/- standard error of the mean (S.E.M) (* p < 0.05; ** p < 0.01; *** p < 0.001).

Brain concentrations of risperidone and 9-hydroxyrisperidone were significantly decreased in all drug transporter inducer groups (Figure 2A-C). Compared to the rifampicin and the dexamethasone group, PCN had the strongest effect on risperidone and 9-hydroxyrisperidone concentrations with a fast wash-out phase (Figure 2A-C). In this group neither risperidone nor 9-hydroxyrisperidone concentrations could be detected 3 h after risperidone injection (Figure 2C). The results of the rifampicin and the dexamethasone groups were quite similar (Figure 2A-B). Quite interestingly, 9-hydroxyrisperidone concentrations increased in all control groups, as expected, because of the metabolism of risperidone to 9-hydroxyrisperidone. However, in all drug transporter inducer groups 9-hydroxyrisperidone brain concentrations showed a maximum value 1 h after risperidone injection without further increase (Figure 2A-C). 

### 3.2. Brain concentrations of risperidone active moiety

**Figure 3:**
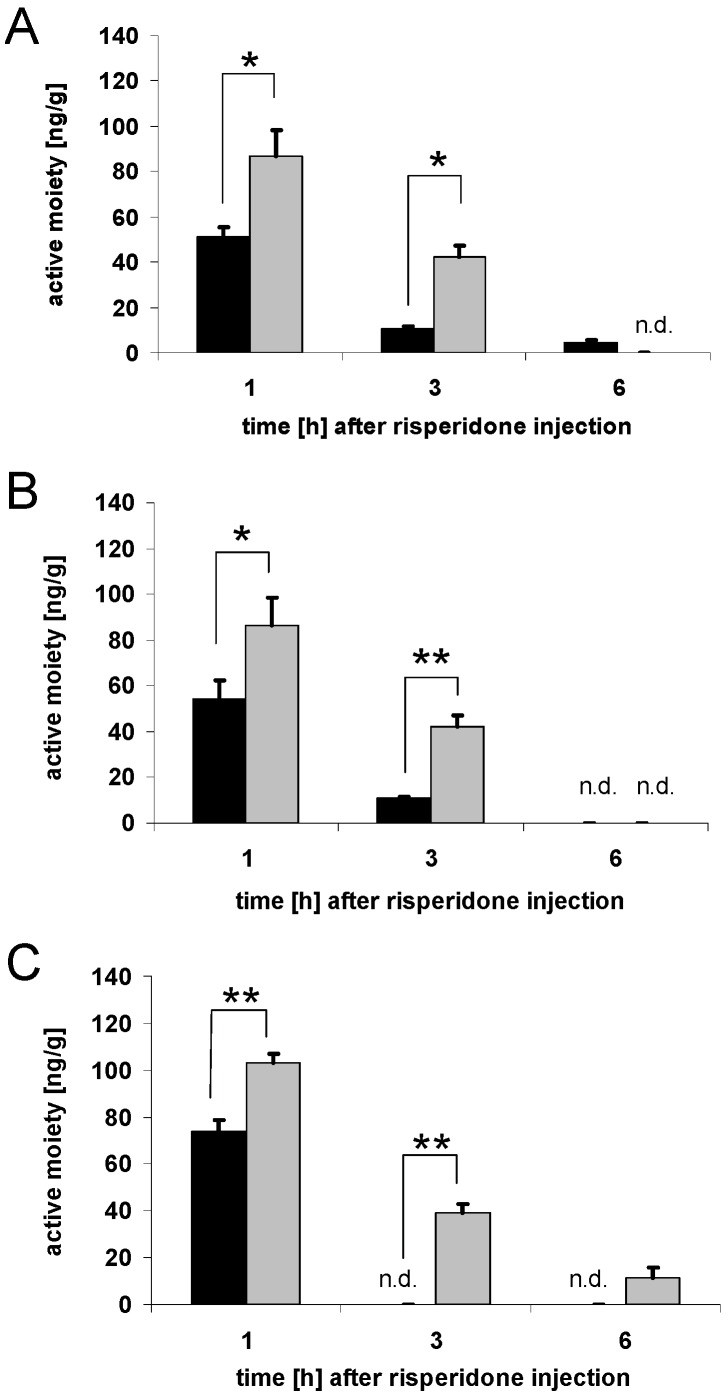
Brain concentrations of risperidone active moiety. Brain concentrations of the active moiety of risperidone and 9-hydroxyrisperidone after drug transporter induction with 10 mg/kg/d rifampicin (A), 50 mg/kg/d dexamethasone (B) or 25 mg/kg/d PCN (C) *vs.* controls for 4 days. Drug transporter-induced mice = black, and controls = grey. Data are mean +/- S.E.M. n.d. = data not detectable due to drug concentrations below limit of quantification, and set to zero for statistical calculations (* p < 0.05; ** p < 0.01).

To summarize the results of the brain concentrations of risperidone and 9-hydroxyrisperidone, the risperidone active moiety was calculated. Mice treated with drug transporter inducing drugs showed decreased brain concentrations of risperidone active moiety compared to the control groups. After four days of rifampicin and dexamethasone treatment, the reduction of the brain concentrations of risperidone active moiety was significant both 1 h and 3 h after risperidone injection (Figure 3A-B). PCN showed a similar effect after 1 h and 3 h with a fast elimination phase (Figure 3C). However, brain concentrations of risperidone active moiety were in some cases below the limit of quantification, and not detectable. This was observed for the PCN group 3 h and 6 h after risperidone injection and the dexamethasone group 6 h after risperidone injection (Figure 3B-C). For controls, only those treated with corn oil as a solvent, for the reasons mentioned above, had detectable levels of risperidone and 9-hydroxyrisperidone 6 h after risperidone injection (Figure 3A-C). 

### 3.3. Serum concentrations of risperidone and 9-hydroxyrisperidone

**Figure 4:**
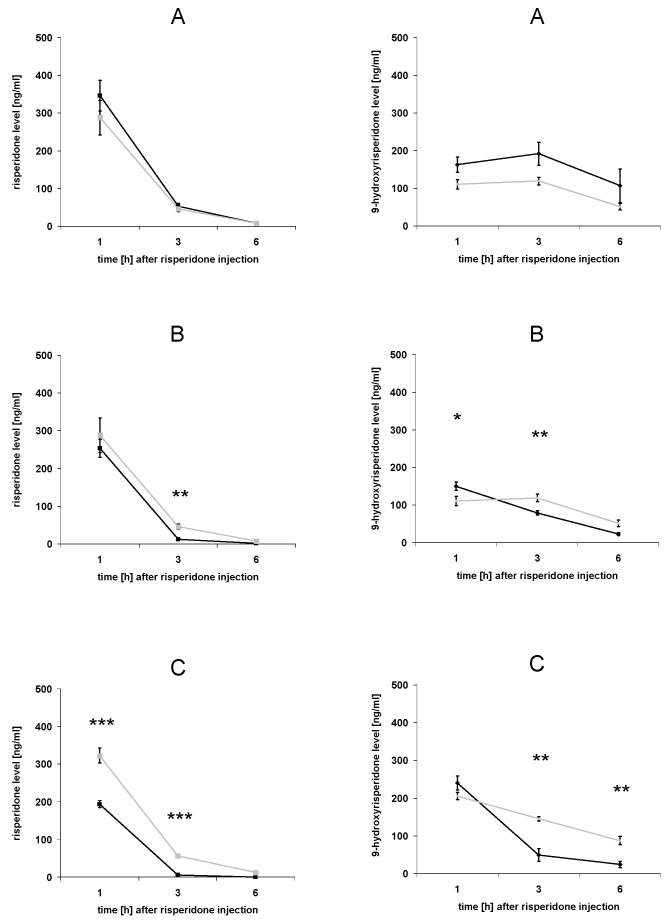
Serum concentrations of risperidone and 9-hydroxyrisperidone. Serum concentrations of risperidone (left side) and 9-hydroxyrisperidone (right side) after drug transporter induction with 10 mg/kg/d rifampicin (A), 50 mg/kg/d dexamethasone (B) or 25 mg/kg/d PCN (C) *vs.* controls for 4 days. Drug transporter induced mice = black lines, controls = grey lines. Data are mean +/- S.E.M. (*p < 0.05; ** p < 0.01; *** p < 0.001).

The results of serum concentrations of risperidone and 9-hydroxyrisperidone differed from those in the brain (Figure 4A-C). Only in the dexamethasone and the PCN group we found significant differences compared to the control group (Figure 4B-C). There were no significant effects in the rifampicin group compared to the control, neither for risperidone concentrations nor for 9-hydroxyrisperidone concentrations (Figure 4A). Interestingly, although not statistically significant, 9-hydroxyrisperidone serum concentrations were higher in the rifampicin group compared to the control group (Figure 4A). In the dexamethasone group, 9-hydroxyrisperidone concentrations were significantly increased 1 h after risperidone injection and significantly decreased 3 h after risperidone injection (Figure 4B). Serum concentrations of 9-hydroxyrisperidone were significantly decreased in the PCN group compared to the control 3 h and 6 h after risperidone injection (Figure 4C). Out of the three drug transporter inducers used, PCN showed the strongest effects on serum concentrations of risperidone and 9-hydroxyrisperidone (Figure 4C).

### 3.4. Serum concentrations of risperidone active moiety

**Figure 5:**
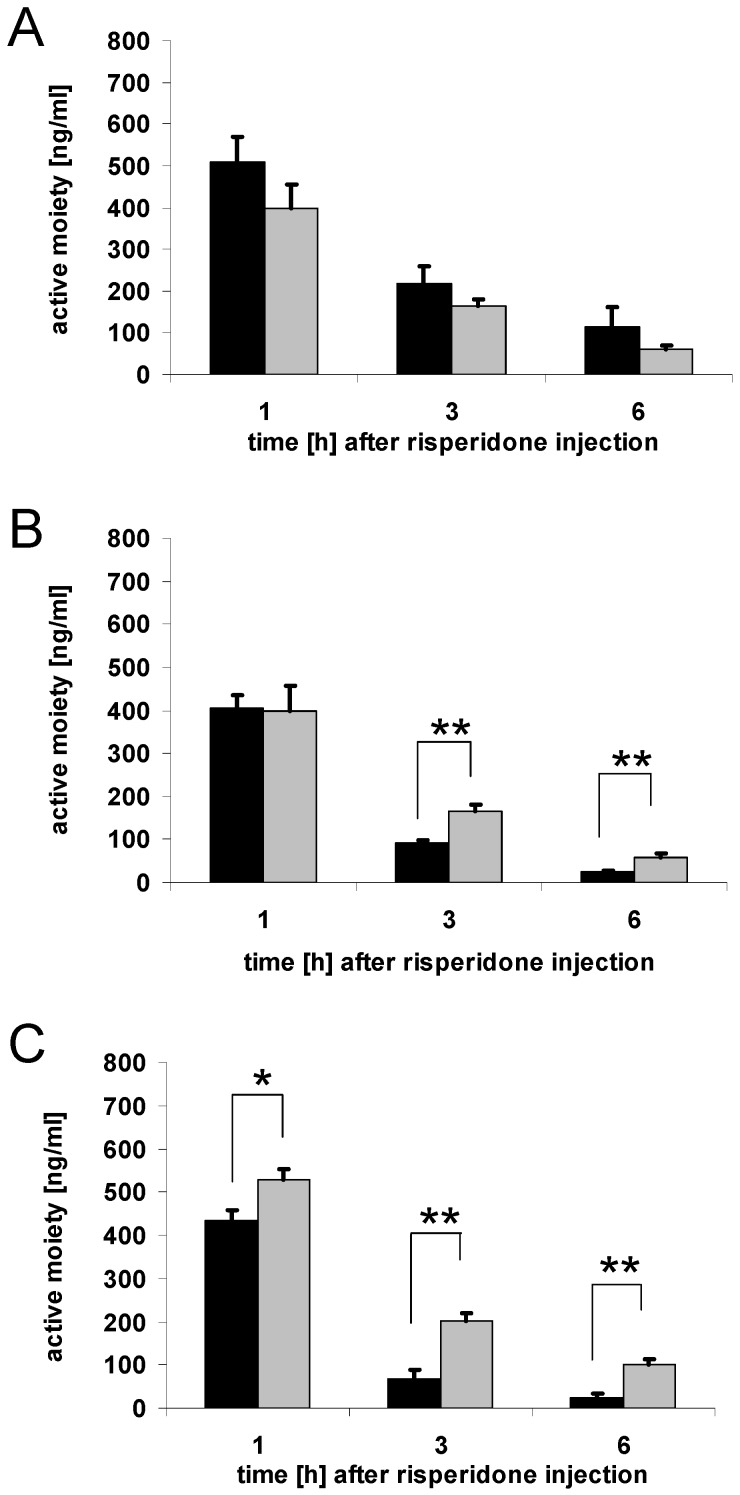
Serum concentrations of risperidone active moiety. Serum concentrations of risperidone active moiety after drug transporter induction with 10 mg/kg/d rifampicin (A), 50 mg/kg/d dexamethasone (B), or 25 mg/kg/d PCN (C) *vs.* controls for 4 days. Drug transporter induced mice = black bars, and controls = grey bars. Data presented are mean +/- S.E.M. (* p < 0.05; ** p < 0.01).

In the case of drug transporter induction with rifampicin, the effect on serum concentrations of risperidone active moiety was not significant 1, 3 or 6 h after risperidone injection (Figure 5A). However, dexamethasone and PCN significantly decreased serum concentrations of risperidone active moiety (Figure 5B-C). Differences were significant after 3 h and 6 h in the dexamethasone group and at all three time points in the PCN group (Figure 5B-C). 

### 3.5. Effects of Drug Transporter Inducers on metabolism

**Figure 6:**
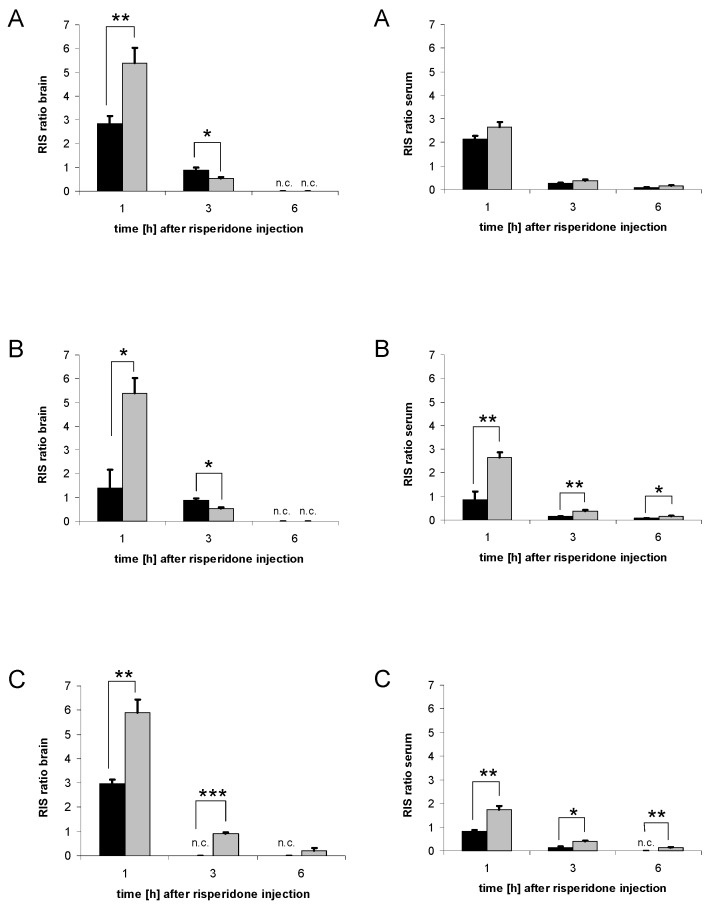
Risperidone/9-hydroxyrisperidone ratio (RIS ratio). RIS ratio of brain (left side) and serum (right side) of mice pre-treated with 10 mg/kg/d rifampicin (A), 50 mg/kg/d dexamethasone (B) or 25 mg/kg/d PCN (C) for 4 days *vs.* controls. Data presented are mean +/- S.E.M. (* p < 0.05; ** p < 0.01; *** p < 0.001). n.c. = data not calculable due to division by zero, and set to zero for statistical calculations.

Drug transporter inducers like rifampicin, dexamethasone and PCN might influence metabolic activity as well. This effect needs to be distinguished from the transport effects to evaluate the potency of drug transporter induction. A decreased RIS ratio indicates an enforced metabolism of risperidone by increased concentrations of the metabolite 9-hydroxyrisperidone. All inducer drugs used in this study affected the RIS ratio (Figure 6A-C). The RIS ratio of brain concentrations in mice pre-treated with rifampicin were significantly decreased 1 h after risperidone injection and significantly increased 3 h after risperidone injection, while RIS ratio in serum showed no significant changes (Figure 6A). These results were similar to the effects found for the dexamethasone group; except for serum levels, which were constantly significantly less than the control group (Figure 6B). In the PCN group, both the RIS ratio of brain and serum were significantly decreased at all three investigated time points (Figure 6C). 

### 3.6. Distribution of risperidone and 9-hydroxyrisperidone

**Figure 7:**
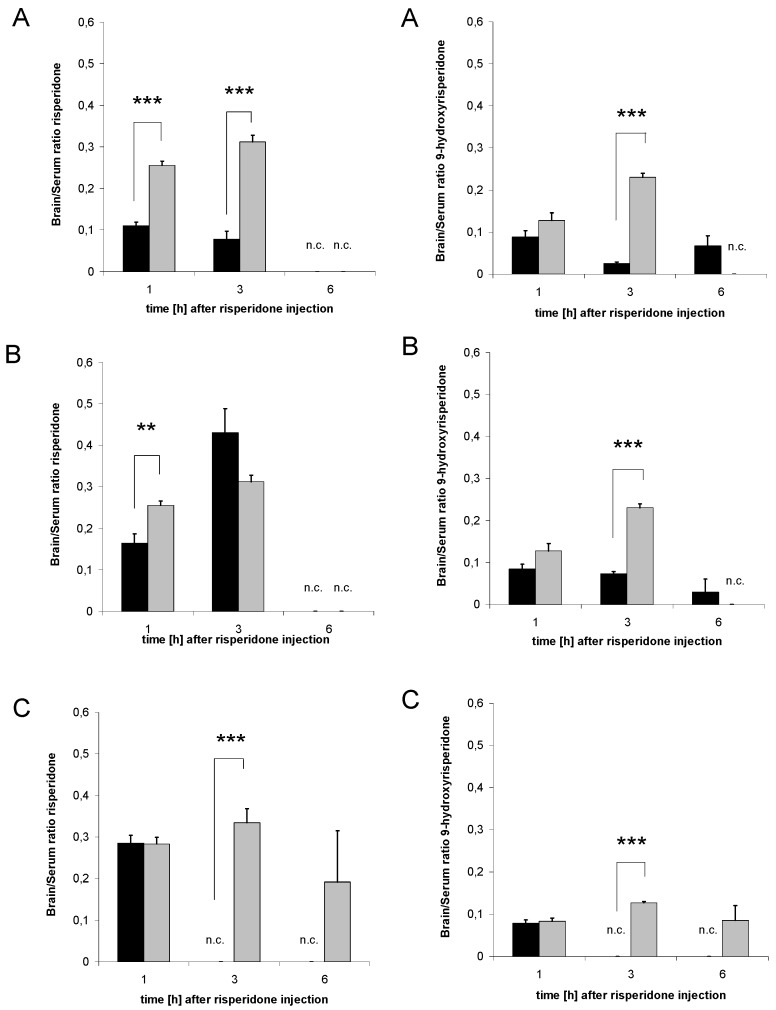
Brain/Serum ratio. Brain/Serum ratio of risperidone (left side) and 9-hydroxyrisperidone (right side) after treatment with 10 mg/kg/d rifampicin (A), 50 mg/kg/d dexamethasone (B) or 25 mg/kg/d PCN (C) *vs.* controls. Data presented are mean +/- S.E.M. (** p < 0.01; *** p < 0.001). n.c. = data not calculable due to division by zero, and set to zero for statistical calculations.

The Brain/Serum ratio gives considerable information about the distribution of drugs in the brain. Brain/Serum ratios at all three investigated time points of mice pre-treated with the drug transporter inducers were compared with the results observed in the controls to show a time-dependent change in the distribution of risperidone and 9-hydroxyrisperidone. A decreased Brain/Serum ratio suggests a reduction of cerebral risperidone and its active metabolite. Brain/Serum ratios of risperidone as well as 9-hydroxyrisperidone were significantly decreased at several time points in the rifampicin, dexamethasone and PCN groups compared to the control (Figure 7A-C).

### 3.7. Distribution of risperidone active moiety

**Figure 8:**
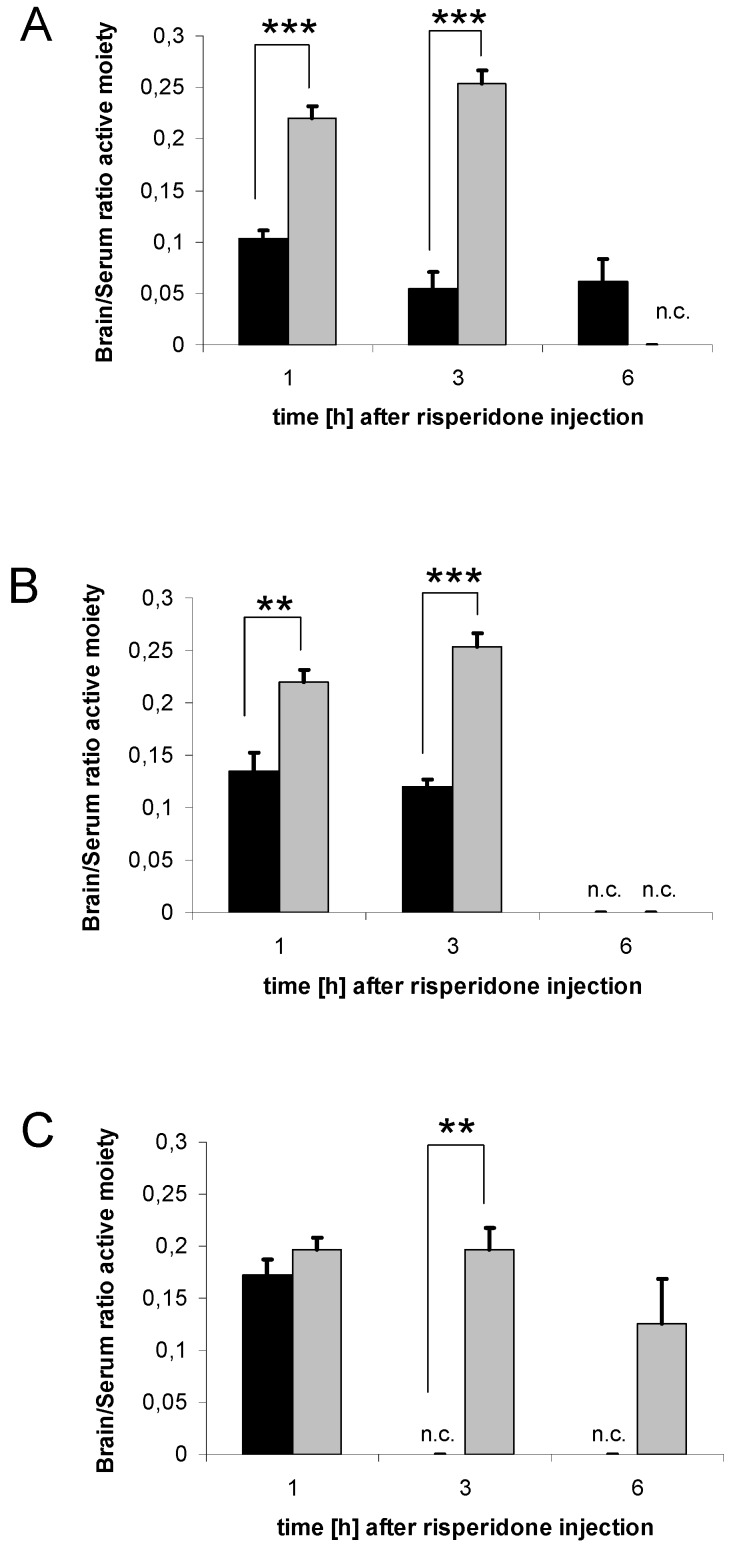
Brain/Serum ratio of risperidone active moiety. Brain/Serum ratio of risperidone active moiety after treatment with 10 mg/kg/d rifampicin (A), 50 mg/kg/d dexamethasone (B) or 25 mg/kg/d PCN (C) *vs*. controls. Data presented are mean +/- S.E.M. (** p < 0.01; *** p < 0.001). n.c. = data not calculable due to division by zero, and set to zero for statistical calculations.

Brain/Serum ratio of risperidone active moiety was significantly decreased in all drug transporter inducer groups (rifampicin, dexamethasone, PCN) compared to the controls at several time points (Figure 8A-C). 

### 3.8. Discussion

The results of the current study have shown that modulation of efflux transporters at the BBB affects the disposition of drugs in the central nervous system in a substantial matter. A supposed increase of drug transporter activity at the BBB was indicated by decreased brain concentrations of the known P-gp substrates risperidone and 9-hydroxyrisperidone. Affinities of risperidone or 9-hydroxyrisperidone to other drug transporters have not yet been investigated, but it is known that P-gp has an outstanding role in the disposition of central active drugs [7,8,9]. Although in humans P-gp deficiency, for example, has not been reported so far, a recent investigation was able to demonstrate that polymorphisms in the human ABCB1 gene, coding for human P-gp, influenced the disposition of risperidone and 9-hydroxyrisperidone [16]. This indicates that modulation of P-gp expression could affect clinical outcome. Pharmacokinetic interactions, due to co-medication, are a common problem in the clinical background. Interactions with P-gp or other drug transporters have not been considered as clinical meaningful so far. The results of our *in vivo* tests in mice showed a clear-cut relationship between supposed up-regulation of drug transporters, e.g. P-gp, and resulting brain concentrations of their substrates, exemplified by the P-gp substrates risperidone and 9-hydroxyrisperidone. This more precisely reflects the physiological conditions at the BBB than a comparison between P-gp wildtype and knockout mouse models. Brain concentrations of risperidone and 9-hydroxyrisperdone were significantly decreased in all drug transporter inducer groups (Figure 2A-C; Figure 3A-C). In particular, 9-hydroxyrisperidone brain concentrations showed differences between inducer and control groups. This partly confirms the hypothesis that induction of drug transporters at the BBB affects 9-hydroxyrisperidone concentrations more than risperidone concentrations. 

As stated in the introduction, 9-hydroxyrisperidone seemed to be a preferred substrate of P-gp. However, this data was collected in *in vivo* studies comparing knockout and wildtype mice. As a matter of fact, in knockout mice, other mechanisms could play a role in the distribution of risperidone or 9-hydroxyrisperidone, because of the P-gp deficiency. In general, we could show that risperidone active moiety was decreased after drug transporter induction, while the difference between transport of risperidone and 9-hydroxyrisperidone was less clear. By shifting the Brain/Serum ratio of the antipsychotic drugs to lower values, a decreased therapeutic effect can be expected, because the brain levels are important for the antipsychotic effect. This is, to our knowledge, the first study that could demonstrate that the co-administration of drugs that are known to modulate drug transporter activity differently affect brain and serum concentrations. Nevertheless, in the clinic, serum concentrations are mostly used as surrogate parameters for brain drug concentrations, which presently can be estimated best via neuro-imaging techniques. Because of the high costs this is not commonly used. As presented, supposed drug transporter up-regulation can significantly reduce both brain and serum concentrations of risperidone active moiety, but brain concentrations are more affected than serum concentrations. In the rifampicin group, for example, serum concentrations of risperidone active moiety did not change significantly while brain concentrations did (Figure. 3A and Figure 5A). This underlies the complexity of pharmacokinetic mechanisms in the brain and that, under certain conditions, serum concentrations do not precisely reflect the situation in the brain. PCN and dexamethasone treatment also decreased serum concentrations of risperidone active moiety in mice, which possibly indicated drug transporter modulation in excreting organs as well (Figure 5B-C).

However, brain and serum concentrations of risperidone active moiety in control mice receiving corn oil as a solvent (PCN group control) were on average 30% and 28% higher, respectively, than the other saline treated controls. This difference between the two solvents used in this study was only significant 6 h after risperidone injection (p = 0.035) for brain concentrations of risperidone active moiety. Differences between saline and corn oil control groups at all other investigated time points were not significant, neither for brain nor for serum concentrations. In conclusion, this factor might be negligible. 

Besides induction of drug transporters, other mechanisms contributing to the present results have to be considered and investigated further. P-gp expression, for example, is linked with PXR activation and species differences in substrate affinities of this transcription factor have been described [17]. PCN selectively activates murine PXR, whereas dexamethasone activates both murine and human PXR [17]. This is inconsistent with our findings. We observed significantly decreased brain concentrations of risperidone and 9-hydroxyrisperidone in mice after treatment with rifampicin. Interestingly, the inducing effect of rifampicin on P-gp expression has previously been shown in another study with rodents [13]. In a clinical study, the impact of rifampicin on risperidone serum levels in humans has been demonstrated [18]. With our study, we could demonstrate that pharmacokinetic interactions with drug transporter inducers can influence brain and serum concentrations of antipsychotic drugs. Up until now, this interaction potential, for whatever reasons, has not attracted the interest of clinicians working in the field of psychiatry. 

Other efflux transporters can also be involved in the transport of risperidone and 9-hydroxyrisperidone. PXR also regulates the expression of the multidrug resistance-associated protein isoform 2 and 3 (MRP3/MRP2), which are two other important transporters at the BBB [19,20]. MRP 2 is highly co-expressed with P-gp [20] and is also, like P-gp and breast cancer resistance protein (BCRP), located at the apical membrane of cerebral endothelial cells [3,21]. MRP2 is possibly able to take over the function of other transport proteins or enhance efflux transport if a substance is a substrate of different efflux transporters. In a study using rats, it was shown that MRP2 deficiency led to P-gp up-regulation [22] and as a conclusion it might very well be possible that both proteins work together in drug efflux at the BBB. P-gp expression can also be modulated by constitutive androstane receptor (CAR) and receptor crosstalk between PXR and CAR can be assumed [23]. Both receptors act as heterodimers with the retinoid X receptor (RXR), for example, and bind to common response elements [23]. There is data demonstrating that nuclear receptor (PXR; CAR) crosstalk can lead to modulation of the expression of multiple metabolic enzymes and transporter proteins in humans [24]. Especially, dexamethasone and PCN could also influence gene transcription pathway through the glucocorticoid receptor (GC) due to their chemical structure. It has previously been shown, that P-gp and BCRP expression is partly mediated by GR activation of dexamethasone, while MRP2 expression showed a GR-independent effect [25].

The induction of P-gp, or other transporters, can also affect the expression of metabolic enzymes. Especially, CYP3A isoenzymes are highly inducible by the drugs used in this study. Previous studies could demonstrate that rifampicin [26], as well as PCN and dexamethasone, induce CYP3A isoenzymes in rodents [27,28,29,30] and humans [31,32]. The RIS ratio gave evidence for effects on the metabolism in our study as well. Enhanced biotransformation by hepatic CYP enzymes can also decrease brain and serum concentrations of risperidone and 9-hydroxyrisperidone by reinforced excretion of the drugs. Cytochrome enzymes in rodents differ from those in humans [11]. Additionally, other metabolic pathways can play a crucial role in biotransformation of risperidone and 9-hydroxyrisperidone in mice. Obviously all drug transporter inducers display impact on metabolism by shifting the RIS ratio. Enforced metabolism of risperidone to 9-hydroxyrisperidone can also interact with drug transport. By increasing concentrations of the preferred P-gp substrate 9-hydroxyrisperidone, brain concentrations of the active moiety were reduced. This was the case in all drug transporter inducer groups (Figure 8A-C). On the other hand, the drug transporter inducers could also be involved in the metabolism of 9-hydroxyrisperidone. As a conclusion, transport and metabolic effects interact in the disposition of risperidone and 9-hydroxyrisperidone. However, as a result, brain and serum concentrations were significantly decreased and that is the important fact for evaluating possible side effects of co-administered drugs. Thus, it is likely that all used substances have multiple effects on the distribution of drugs, which needs to be better understood. 

By calculating Brain/Serum ratios, we wanted to estimate the amount of risperidone and 9-hydroxyrisperidone reaching the CNS. These ratios give considerable information about whether rifampicin, dexamethasone or PCN change distribution and efflux of the antipsychotic drugs in the brain. The results of our study revealed that all drug transporter inducer groups decreased brain distribution of risperidone and 9-hydroxyrisperidone and the risperidone active moiety of risperidone and 9-hydroxyrisperidone, although PCN had the strongest effects on brain concentrations of risperidone active moiety (Figure7A-C). Brain/Serum ratio of the active moiety clearly showed that brain disposition of risperidone and 9-hydroxyrisperidone is influenced by supposable drug transporter induction (Figure 8A-C). A pharmacokinetic interaction due to drug transporter modulation in this range is likely to influence the efficacy of drugs. However, it remains unclear if the effect on PXR or possible effects on other transcription factors, e.g. constitutive androstane receptor (CAR) or glucocorticoid receptor (GR), are responsible for the results observed in the present study.

## 4. Conclusions

In conclusion, by using risperidone and 9-hydroxyrisperidone concentrations as a tool we were able to evaluate the functional role of drug transporter modulation in BBB transport. Despite the fact that we could not definitely relate all investigated effects to drug transport, there is clear evidence that brain and serum concentrations of risperidone and its active metabolite can be influenced by co-administered drugs. By decreasing brain and serum concentrations of risperidone and its active metabolite 9-hydroxyrisperidone, a reduced pharmacodynamic effect can be expected. The decreased brain concentrations of risperidone and 9-hydroxyrisperidone in mice exemplified the physiological function of drug transporters for disposition of CNS active drugs. Further studies are needed to clarify the involvement of other BBB transporters in the distribution of these antipsychotic drugs to evaluate other possible interactions contributing to the failure of antipsychotic therapy. 
